# CeO_2_ nanoparticle dose and exposure modulate soybean development and plant-mediated responses in root-associated bacterial communities

**DOI:** 10.1038/s41598-024-60344-8

**Published:** 2024-05-03

**Authors:** Jay R. Reichman, Matthew R. Slattery, Mark G. Johnson, Christian P. Andersen, Stacey L. Harper

**Affiliations:** 1 Pacific Ecological Systems Division, Office of Research and Development, US Environmental Protection Agency, Corvallis, OR 97333 USA; 2 Department of Environmental and Molecular Toxicology, Oregon State University, Corvallis, OR 97331 USA; 3 Department of Botany and Plant Pathology, Oregon State University, Corvallis, OR 97331 USA; 4 School of Chemical, Biological and Environmental Engineering, Oregon State University, Corvallis, OR 97331 USA

**Keywords:** Nanoscience and technology, Ecotoxicology, Plant sciences, Plant stress responses, Plant symbiosis, Environmental microbiology

## Abstract

Agricultural soils are increasingly undergoing inadvertent and purposeful exposures to engineered CeO_2_ nanoparticles (NPs), which can impact crops and root-associated microbial communities. However, interactions between NP concentration and exposure duration on plant-mediated responses of root-associated bacterial communities are not well understood. Soybeans seedlings were grown in soil with uncoated NPs added at concentrations of 0, 1 or 100 mg kg^−1^. Total soil exposure durations were either 190 days, starting 106 days before planting or 84 days with NP amendments coinciding with planting. We assessed plant development, bacterial diversity, differential abundance and inferred functional changes across rhizosphere, rhizoplane, and root tissue compartments. Plant non-monotonic dose responses were mirrored in bacterial communities. Most notably, effects were magnified in the rhizoplane under low-dose, short-exposures. Enriched metabolic pathways were primarily related to biosynthesis and degradation/utilization/assimilation, rather than responses to metals or oxidative stress. Our results indicate that plant-mediated bacterial responses were greater than direct NP impacts. Also, we identify needs for modeling non-monotonic legume stress responses that account for coinfection with mutualistic and parasitic bacteroids. Our findings provide new insights regarding effects of applications of soil amendments such as biosolids containing NPs or nano-enabled formulations used in cultivation of legumes and other crops.

## Introduction

Agricultural soils undergo inadvertent and purposeful exposures to engineered cerium oxide nanoparticles (CeO_2_ NPs), which can impact crops and root-associated microbial communities. Thousands of metric tons of CeO_2_ NPs are produced each year for use in fuel cells, electronic and optical devices, chemical planarization, medical products, coatings, polishing agents, and fuel catalysts^1–7^. Most often, CeO_2_ NPs enter and accumulate in agricultural soils from application of wastewater treatment plant biosolids, wastewater irrigation and via aerial deposition^8–12^. Biosolids application and wastewater irrigation are the most substantial sources of CeO_2_ NPs in agronomic habitats, with 90% of the NPs introduced to wastewater treatment plants remaining in the sludge after processing^13,14^. Aerial deposition of emissions from combusted diesel fuel containing CeO_2_ NP catalysts can also potentially raise soil concentrations by as much as 1 mg kg^−1^ at 96 m downwind from highways^9,15^. In addition, there have been recent increased developments of CeO_2_ NPs in soil amendments and fertilizer formulations that are intended to protect crops from abiotic stressors and improve water use efficiency^16–19^.

Crops and other terrestrial plants are dependent on root exudates (organic acids, cellulosic materials, and amino acids) to promote the development of root-associated microbial communities nested within the rhizosphere^20^. This zone contains a high concentration of bacteria and fungi that can be critical for nutrient cycling, establishing symbiotic relationships, providing protection from herbivores and pathogens, and ultimately facilitating plant growth^21,22^. In soybeans, root nodules containing bacterial symbionts are responsible for an estimated 77% of the nitrogen fixed by leguminous crops^23^. The broader rhizosphere community can be sub-divided into those microbes living in the apoplastic space between root cells (endorhizosphere), those associated with the outside of the root including mucilage (rhizoplane), and those in the zone linking the rhizoplane to the bulk soil (ectorhizosphere)^20^. The effects of CeO_2_ NPs on critical interactions between plants and bacterial communities in the root, rhizoplane and outer rhizosphere compartments are not well understood, particularly for important crops such as soybeans.

Previous research indicates that when agricultural soils are exposed to engineered CeO_2_ NPs shortly prior to planting crops, shifts in root-associated bacterial communities are indirectly influenced by plant responses. Priester and colleagues^24^ amended agricultural soils with CeO_2_ NPs at 100—1000 mg kg^−1^, 24 h before planting soybean seedlings. After 48 days, they found non-monotonic dose-responses in plant growth, with the CeO_2_ NP low-dose exerting the most impacts, which included stunted plant growth. In contrast, pod number increased with increasing dose while N_2_ fixation by symbionts in root nodules decreased. These effects were associated with Ce uptake from soil into the roots, but which were not found to measurably translocate to aboveground biomass in that study. Follow-on research demonstrated that soybean exposure to 100 and 500 mg kg^−1^ CeO_2_ NP soil resulted in bioaccumulation of Ce in roots, nodules, and leaves. Then, aboveground plant production became impaired by oxidative leaf damage from peroxidized lipids and reactive oxygen species, which corresponded to lower root nodule N_2_ fixation potential. The authors concluded that soybeans grown with CeO_2_ NPs apparently protected aboveground biomass at the expense of belowground symbioses^25^. Computational simulations of soybean non-monotonic plant responses to CeO_2_ NP exposure are discussed below^26^.

In additional studies with soybeans, Ge and colleagues^27^ showed that the same 48-day CeO_2_ NP exposures used in the earlier work^24^ did not affect bacterial communities in unplanted soils. However, a 100 mg kg^−1^ CeO_2_ NP concentration was associated with alteration of soil bacterial communities when plants were present. This important finding linked low CeO_2_ NP dose to potential changes in the quantity and composition of plant root exudates, which then interactively promoted effects to microbes in the soil.

The conceptual framework whereby CeO_2_ NP impacts to soil microbiota can be indirect and influenced by feedback from plants is supported by other experimental results from studies with and without plants as well. For example, experiments using synchrotron spectroscopy showed that multiple plant species can augment CeO_2_ NP reduction in soil and hydroponic media surrounding roots^28–36^. These biotransformations were attributed to properties of root exudates of the plants and were associated with species-specific differential Ce uptake into the roots. Also, when canola plants were grown for 30 days in soil spiked with three differently designed CeO_2_ NPs at 1 mg kg^−1^ at the time of planting^37^, an impact gradient on microbial activity and bacterial community structure was identified, with maximum effects near the root surface compared to rhizosphere or bulk soil. In other experiments run without plants, 42-day soil exposures to CeO_2_ NPs were reported to have limited impacts on bacterial community structure^38^.

Underlying the plant-mediated bacterial community responses to nanoparticle exposure are the time-dependent transformations that occur when NPs enter soils. While CeO_2_ NPs are highly stable in a wide variety of soils^39^, NPs can still be transformed through a variety of abiotic and biotic processes. Transformations like these have the potential to alter the fate, transport, and toxicity of nanomaterials^12,40–44^. For example, it has been reported that properties of soils where CeO_2_ NPs are deposited can antagonistically influence the phytoavailability of NPs to tomato and fescue^45^. In addition, soil bacteria reportedly secrete siderophores and extracellular polymeric substances that can influence nanoparticle characteristics and reactivity^46–48^. Many NP transformations in complex environmental media like soils are gradual and not easily predicted. Real world environmental exposures to NPs are likely to be chronic, at low concentrations, and therefore, relatively slow. Little is known about the rates of these transformations under relevant conditions^43^.

One of the few studies on the temporal changes of the properties of CeO_2_ NPs in the environment, considered effects on fractionation of NPs at 1000 mg kg^−1^ in a silty loam soil and their bioavailability to radish^49^. The authors found that 7 months of aging did not affect the partitioning of Ce among soil fractions. However, soil with aged NPs had 40.5% higher concentration of Ce^3+^ than soil with fresh NPs. Also, radish shoots grown in soil with the longer interaction time had 87% higher Ce concentration than those from freshly exposed soil. In another case, a 25-month outdoor lysimeter study investigated the translocation, biological impact, and transformation of CeO_2_ NPs applied via spiked sewage sludge to soils at 10 or 50 mg kg^−1^ 3 weeks before initiating a 24-month crop rotation^50^. There was no vertical translocation of Ce into deeper soil or percolating water. Also, the results implied that there was low bioavailability of CeO_2_ NPs in soil.

The objectives of this study were to address the following questions. Do the interactions between CeO_2_ NP soil concentration and exposure duration affect soybean development? Do interactions between NP dose and exposure change communities of bacteria in root, rhizoplane, and rhizosphere compartments? If so, is there evidence for direct effects and/or plant-mediated responses in the root-associated bacterial communities? Here, the NP doses span expected environmentally relevant concentrations in agricultural soils. Exposure durations were either 190 days, which provided ~ 3 months of CeO_2_ NP-soil interaction time prior to planting or 84 days with soil exposure coinciding with the presence of plants. Above and belowground developmental endpoints were measured to assess impacts to the soybean plants. In addition, we conducted fine-scale sampling of root, rhizoplane, and rhizosphere bacterial communities for 16S rRNA gene amplicon sequencing and inferred metabolic pathway enrichment analyses to test for treatment differences that were associated with those compartments.

## Methods

The materials and methods below were modified from^51,52^. A generalized timeline for the experiments is shown in Table 1. Details follow below.

**Table 1:** Timeline for soil preparation, inoculation, CeO_2_ NP exposures, germination, planting, and sampling.

Day	Event
0	Soil was mixed with rhizobia and distributed into 5 tubs (1 tub/experimental group)
	CeO_2_ NPs were only mixed into high and low-dose tubs to be used for the 190-day exposures
	All tubs were watered to ~ 75% of their field capacity
	All tubs were transfered to a growth chamber with no lights at 25°C
99	Soybean seed germinations were started in peat pellets
105	Soil tubs were removed from the growth chamber and mixed
	CeO_2_ NPs were only mixed into high and low-dose tubs to be used for the 84-day exposures
	Pots were filled with 2.5 kg soil/pot
106	Pots were planted with 1 seedling/pot (N = 10/experimental group)
	Pots were transferred to a greenhouse with ambient light at 20°C
	Pots were watered weekly from below to ~ 85% field capacity
190	Plants were harvested
	Above and belowground parameters were measured
	Rhizosphere, rhizoplane, and root compartments were sampled for metagenomic DNA to be used in bacterial 16S rRNA gene amplicon sequencing (N = 4/experimental group/compartment)

### Nanoparticles

Uncoated CeO_2_ NPs were purchased from Sigma Aldrich (St Louis, MO, USA; batch number MKCB0040V) in powder form with a primary particle size of 16 nm, verified by the manufacturer using X-ray diffraction and Brunauer–Emmett–Teller analysis. For soil amendment working stocks, CeO_2_ NPs were suspended in deionized (DI) water at either 125 mg L^−1^ (for low concentration soils) or 12,500 mg L^−1^ (for high concentration soils). Then, the suspensions were sonicated for 5 min at 100% intensity with a VCX 750 Vibra-Cell sonicator (Sonics and Materials Inc., Newton, CT, USA) with a cup-horn style high intensity probe in a recirculating bath.

### Soil preparation

A common coarse-textured floodplain soil (Newberg soil series, coarse-loamy, mixed, super active, mesic Fluventic Haploxerolls) from the Willamette Valley of Oregon, USA was collected during the annual dry period, air-dried, sieved (6.35 mm sieve) and homogenized. The soil was 63.8% sand, 20.1% silt, and 16.1% clay, which is a sandy loam texture. It had a pH of 6.6 with 0.5% C, as determined by the Oregon State University Central Analytical Laboratory. For these characteristics, the soil was similar to that used in previous studies^24,27,53,54^. Additional properties are shown in the Supplementary Information Table S1. On Day-0, 25 kg aliquots of soil were added to individual plastic tubs for each of the 5 experimental groups: control, low 84-day, high 84-day, low 190-day, and high 190-day (described below). All soil tubs were inoculated with a commercially prepared suspension of *Bradyrhizobium japonicum* specialized for symbioses in legumes (America’s Best Soybean Inoculant® batch #2G18210) from Advanced Biological Marketing, Van Wert, Ohio, USA. The inoculation was at 2× the field rate per the manufacturer’s instructions resulting in ≥ 2.4 × 10^8^ CFU m^2^ soil surface. The soil in each tub was then thoroughly mixed.

### Soil exposures

Total soil exposure durations were either 190 days, starting 106 days before planting or 84 days coinciding with presence of plants. After the *B. japonicum* inoculations on Day-0, CeO_2_ NPs were added to just the tubs for the two 190-day treatments, at low (1 mg kg^−1^ dry soil) and high (100 mg kg^−1^) concentrations. All tubs were watered to ~ 75% of their field capacity, individually homogenized with a clean shovel, and then placed in a temperature-controlled incubation chamber set to 25 °C and relative humidity at 70%.

At Day-105, the soil tubs were removed from the growth chamber, and CeO_2_ NPs were added only to the 84-day treatments at the same low and high concentrations. Then, all tubs were watered to 85% of field capacity and homogenized a second time, at which point the soils were ready for transfer to 20 cm diameter pots. Each pot contained 3 cm of crushed gravel at its base, a polyethylene grow bag with 12 holes at the bottom for drainage and bottom watering, and 2.5 kg of prepared soil in the bag. Each of the 5 experimental groups had 10 replicates.

### Soybean cultivation

Soybean seeds (*Glycine max,* Beer Friend variety, lot #3301001) were purchased from Victory Seeds, Molalla, Oregon, USA. This variety was selected for its rapid time to maturity. On Day-99, an excess of 200 seeds were rinsed with deionized water before placement into peat starter pellets in a greenhouse under natural lighting. On Day-106, 50 healthy seedlings at the same developmental stage were selected and individually transfered to prepared pots in the greenhouse. Soybeans were watered once per week from below by submerging the base of pots in individual trays containing 10 cm of water until the topsoil was visibly moistened. The positions of pots on the greenhouse bench were randomized at each watering event. Soybeans were harvested for processing after 84 days of growth, at which point plants were in reproductive stage R6/R7 (full seed/beginning maturity).

At harvest on Day-190, the tissues were separated into stems, leaves, pods, beans, roots, and nodules. Stem lengths were measured, then pods, beans, and nodules were counted. Nodules were sliced open to check for red or pink coloration, indicating active fixation of atmospheric nitrogen^55^. Also, a 5 cm section of root material with associated soil was excised from each root mass for microbial compartment sampling and metagenomic DNA extraction. Aboveground tissues and nodules were placed in paper bags and kept in a drying oven for 7 days at 105 °C, then weighed.

### Rhizosphere, rhizoplane, and root compartment sampling

To generate spatially distinct bacterial community data, we adapted a method from^56^. Briefly, 1 g of root material (including loosely bound soil) was placed in a 50 mL falcon tube with 40 mL of phosphate buffered saline (PBS) solution (8 g L^−1^ NaCl, 0.2 g L^−1^ KCl, 1.42 g L^−1^ Na_2_HPO_4_, 0.24 g L^−1^ KH_2_PO_4_). Sterile forceps were used to stir the root material in the vial, releasing some of the soil into solution representing the “rhizosphere” compartment. The mixtures were transferred to microfuge tubes and centrifuged at 10,000 g for 30 s, then the liquid was aspirated leaving the soil pellet. Next, the roots from the previous step were placed in a new falcon tube with 40 mL PBS and sonicated for 45 s in a bath ultrasonicator, releasing more tightly bound soil from root surfaces representing the “rhizoplane” compartment. The soil pellet was collected as before. Finally, the root tissue was placed in 40 mL of fresh PBS and sonicated for 45 s, placed in fresh PBS buffer and sonicated for 45 s again, then removed and 0.250 g of tissue was placed in a dry falcon tube representing the “root” compartment. All microbial compartment samples were frozen at –20 °C until DNA extractions were performed.

### DNA extractions and amplicon sequence library preparations

Metagenomic DNA was extracted from microbial compartment samples (N = 4/experimental group/compartment) using PowerSoil Kits per the manufacturer's instructions (Qiagen). The DNA was quantified with a Qubit 3.0 (Thermo Fisher). Bacterial amplicon sequencing libraries were prepared based on the Illumina amplicon‐seq protocol^57^. For amplification of 16S rRNA gene V3/V4 hypervariable regions from prokaryotes, the amplicon polymerase chain reaction (PCR) step was performed with S‐D‐Bact‐0341‐b‐S‐17 and S‐D‐Bact‐0785‐a‐A‐21 primers^58^ that had Illumina overhang adaptors attached^57^. Primers were synthesized by Eurofins Genetics. Dual index PCRs utilized Nextera XT Index Kit v2 Set A (Illumina). Amplicon and index cocktails were prepared with 2X KAPA HiFi HotStart ReadyMix (KAPA Biosystems), and all sets of PCRs included no template controls to check for reagent contamination. No amplifiable DNA was detected in any of these contamination controls. PCR products were purified with Agencourt AMPure XP Beads (Beckman). Amplicon size distributions were determined with a Bioanalyzer 2100 using DNA 1000 chips (Agilent). Paired‐end sequencing data were collected on an Illumina MiSeq using MiSeq Reagent Kits v3, 600 cycles (Illumina) per the manufacturer's protocol.

### Data analyses and statistics

Plant phenotypic data was log transformed and the R package Stats v 4.2.0 was used for two‐way analysis of variance (ANOVA) comparisons. Then, Agricolae v1.3-5 was used to perform HSD post hoc analysis. The QIIME 2 Core 2021.2 package was used for quality control, diversity analyses (sampling depth = 35,000), ordinations, visualizations, and statistical analyses of amplicon sequencing data^59^. Specific QIIME 2 plugins applied during the present study included Cutadapt for trimming Illumina adaptors from reads^60^; DADA2 to denoise trimmed reads into amplicon sequence variants (ASVs; proxies for species;^61^); MAFFT for sequence alignments^62^; FastTree 2 to generate phylogenetic trees^63^; *q2‐feature‐classifiers*^64^ using the GreenGenes 13‐8‐99 classifier^65^ for taxonomic identification of 16S ASVs; *qiime composition ANCOM*^66^ used to quantify differential abundance of ASVs with frequencies of 175 or more and a corrected false discovery rate (FDR) = 0.05; and *qiime diversity bioenv* to assess correlations among beta diversity distances, exposure dose, and duration^67^.

Inferred functional analyses of differentially abundant ASVs were performed with PICRUSt2, which is an algorithm to predict bacterial metabolic pathway abundances from 16S marker sequences and counts^68^. Paired groups of enriched MetaCyc pathways were compared in STAMP v2.1.3^69^ based on Welch’s two-way t-test, with Benjamini–Hochberg multiple test correction, Confidence Interval = 0.95. Pathways were filtered for q ≤ 0.05 and to remove those with effect size differences < 3.

### Experiments and field studies on plants

The Study complies with local and national guidelines and regulations.

## Results and discussion

### Soybean developmental impacts

**Figure 1:**
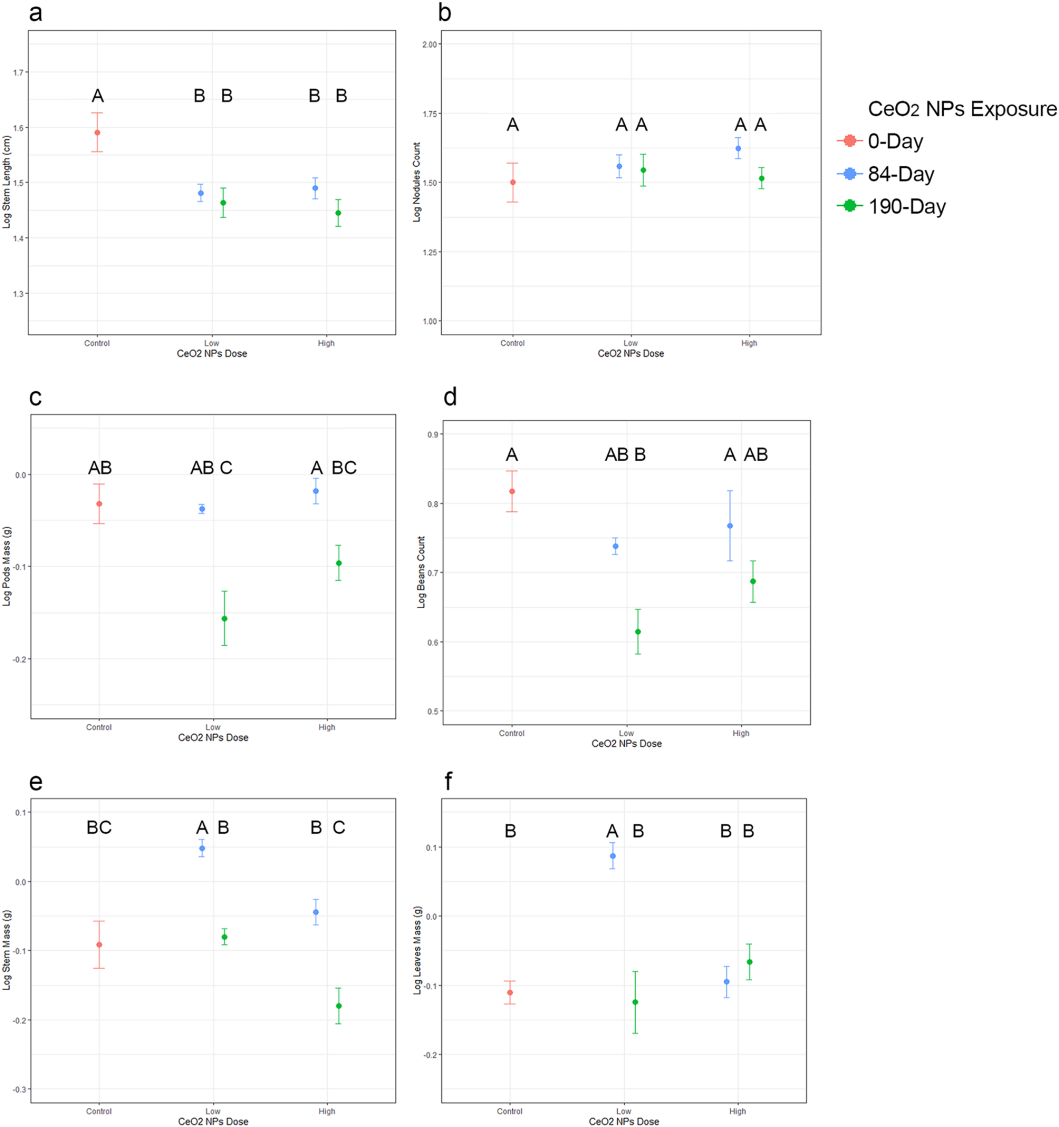
Development of soybean plants exposed to CeO_2_ NPs via soil. Mean log stem length was shorter for plants in all treated groups compared to controls (**a**). However, there were no significant differences in log nodule counts (**b**). There were non-monotonic decreases for pod mass and bean count at the low 1mg kg^−1^ dose after 84 Days of exposure (**c** and **d** respectively). Also, log stem and leaf mass had non-monotonic increases under the same conditions (**e** and **f** respectively). Upper case letters indicate significant differences among means based on two-way ANOVA with HSD post hoc analysis (corrected *p* ≤ 0.05). Error bars show ± 1 SE (N = 10).

Our results show that both CeO_2_ NP dose and exposure duration affect soybean development (Fig. 1). This was found at our environmentally relevant 1 mg kg^−1^ low-dose, especially where the shorter exposure duration coincided with planting. At harvest all significant changes in treated plants occurred above ground, with 56% of instances of significance following 84-day exposures and 67% at low-dose concentrations. Supplementary Fig. S1 shows the distribution of all the plant phenotypic log transformed data for each of the 10 parameters we measured along with indications of significant differences among the means. Given that our high-dose corresponded to the concentration that impacted soybeans grown in similar soil in a previous study^24^, we expected our results to be comparable.

There was significantly stunted stem length under all treatments. Also, non-monotonic decreases in dry pod mass and bean count were observed with the low-dose 190-day exposures. In contrast, stem and leaf mass had non-monotonic increases in low-dose short exposure samples. For pod count, the was a positive dose–response following 84-day exposures, with a significant increase at the 100 mg kg^−1^ concentration. Nodule counts and mass were not affected by the treatments.

The results indicate that soybeans were sensitive to both the environmentally relevant 1 mg kg^−1^ CeO_2_ NP soil concentration and the 100 mg kg^−1^ high-dose. Furthermore, exposure to cerium had the broadest impact on plant development after the low-dose 84-day exposures, where the CeO_2_ NPs had the least abiotic/biotic interaction time within the soil before planting. Our quantitative results were similar to those found by Priester and colleagues^24^ in 3 regards: (1) the stunting of plant growth, (2) non-monotonic developmental responses, and (3) the positive dose–response for pod counts. The reasons for the tissue specific non-monotonic dose-responses associated with the different NP exposure durations observed here are unclear. Although N_2_ fixation was not measure directly, our qualitative assessment of root nodules found no indication of altered function in treated plants compared to controls. This was congruent with the previous findings that 100 mg kg^−1^ of CeO_2_ NPs did not significantly change N_2_ fixation by symbionts^24^. While increasing Ce concentration in soybean nodules was found to be negatively correlated with N_2_ fixation in a previous study^25^, such effects would be more likely at higher soil CeO_2_ NP doses than were used here.

### Alpha and beta diversity metrics for bacterial communities

**Figure 2:**
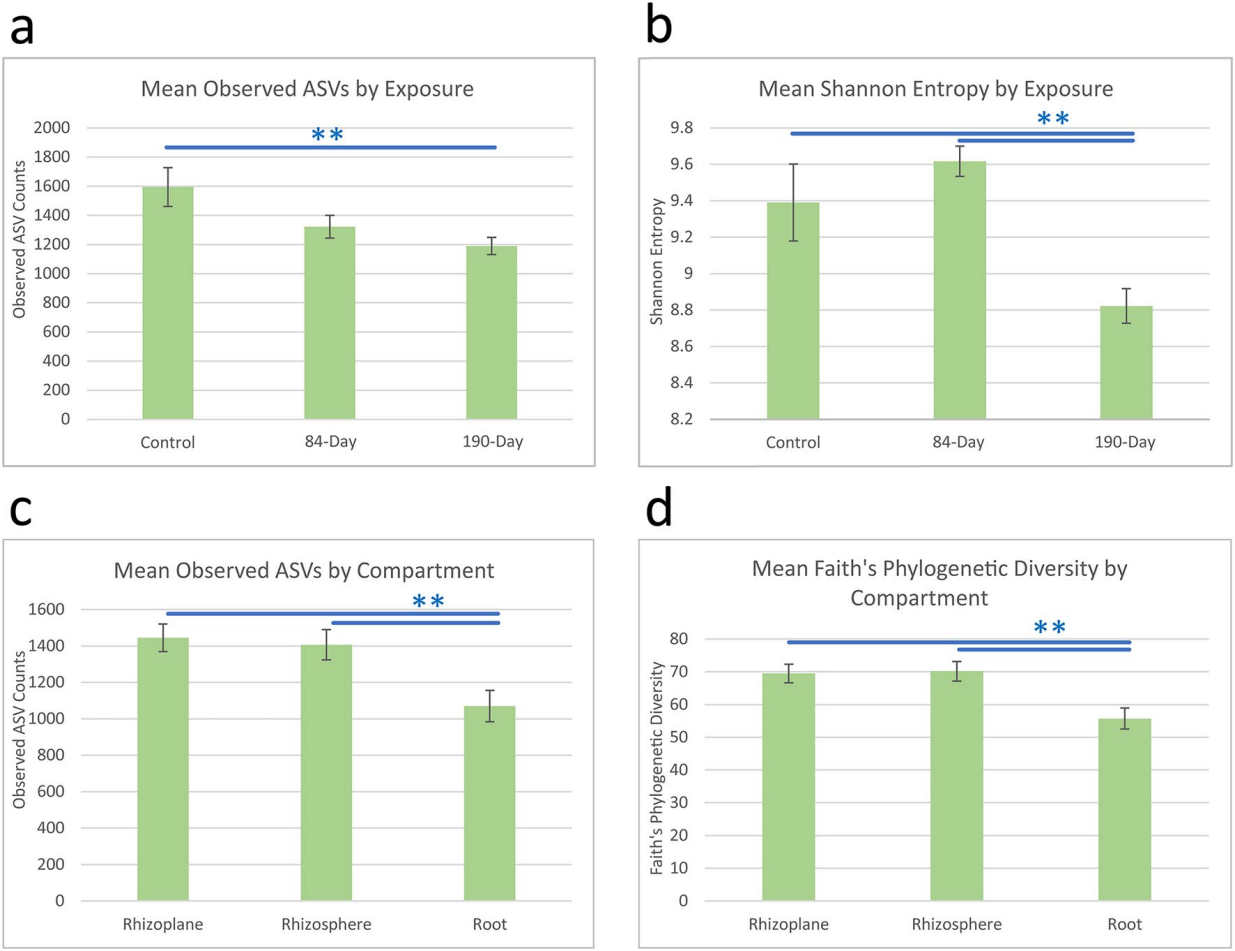
Soil bacterial communities exposed to CeO_2_ NPs for 190-days had fewer mean observed ASVs than controls (**a**) and lower mean Shannon entropy than control and samples exposed for 84-days (**b**). Whereas the root compartment had lower observed ASVs (**c**) and Faith’s phylogenetic diversity (**d**) than those from the rhizosphere or rhizoplane. Error bars show ± 1 SE. ** indicates Krustal–Wallis q-values ≤ 0.05.

Bioinformatic analyses of our 16S rRNA gene ASV data, including group significance tests, identified that exposure duration, but not dose, significantly affected alpha diversity of bacterial communities (q-values ≤ 0.05, Fig. 2a,b, Supplementary Table S2). Samples from 190-day NP exposures had reduced mean observed ASVs and Shannon entropy compared to controls. Observed ASV counts reflect the species richness, the number of different taxa detected in the samples. The related Shannon entropy gives a measure of species diversity based on how evenly proportions of taxa are distributed in samples. Not surprisingly, samples from the root compartment had lower observed ASVs and Faith’s phylogenetic diversity than those from the rhizosphere or rhizoplane (Fig. 2c,d, Supplementary Table S2). Reduced phylogenetic diversity indicates fewer dissimilar ASVs in samples. The expectation was that the number and types of bacteria that can potentially enter root tissue would be lower than those present within the broader soil communities extending out to the rhizosphere. Those able to enter roots and persist would generally be potential symbionts, parasites, or pathogens.

Our results suggest it is possible that under the longer 190-day exposure scenario, 3-months of pre-planting CeO_2_ NP-soil interactions was sufficient time to eliminate some sensitive microbial taxa and to shift the alpha diversity, which could persist throughout the remainder of the experiments. In other studies, short-term CeO_2_ NP exposures (42–48 days) in the absence of plants did not significantly affect soil bacterial communities^27,38^. It is also possible that biotic and abiotic dynamics may have reduced, but not eliminated, the NP toxicity to plants during the extended incubation, as well. For example, a portion of NP surfaces may have become partially covered by compounds expressed by bacteria and/or organic matter within the soil. Lowered NP toxicity would generally agree with our plant developmental results presented above. Reduced stress in the 190-day exposed samples compared to the 84-day samples may have also resulted in different root exudate profiles and plant-mediated impacts on microbial communities within the compartments.

**Figure 3:**
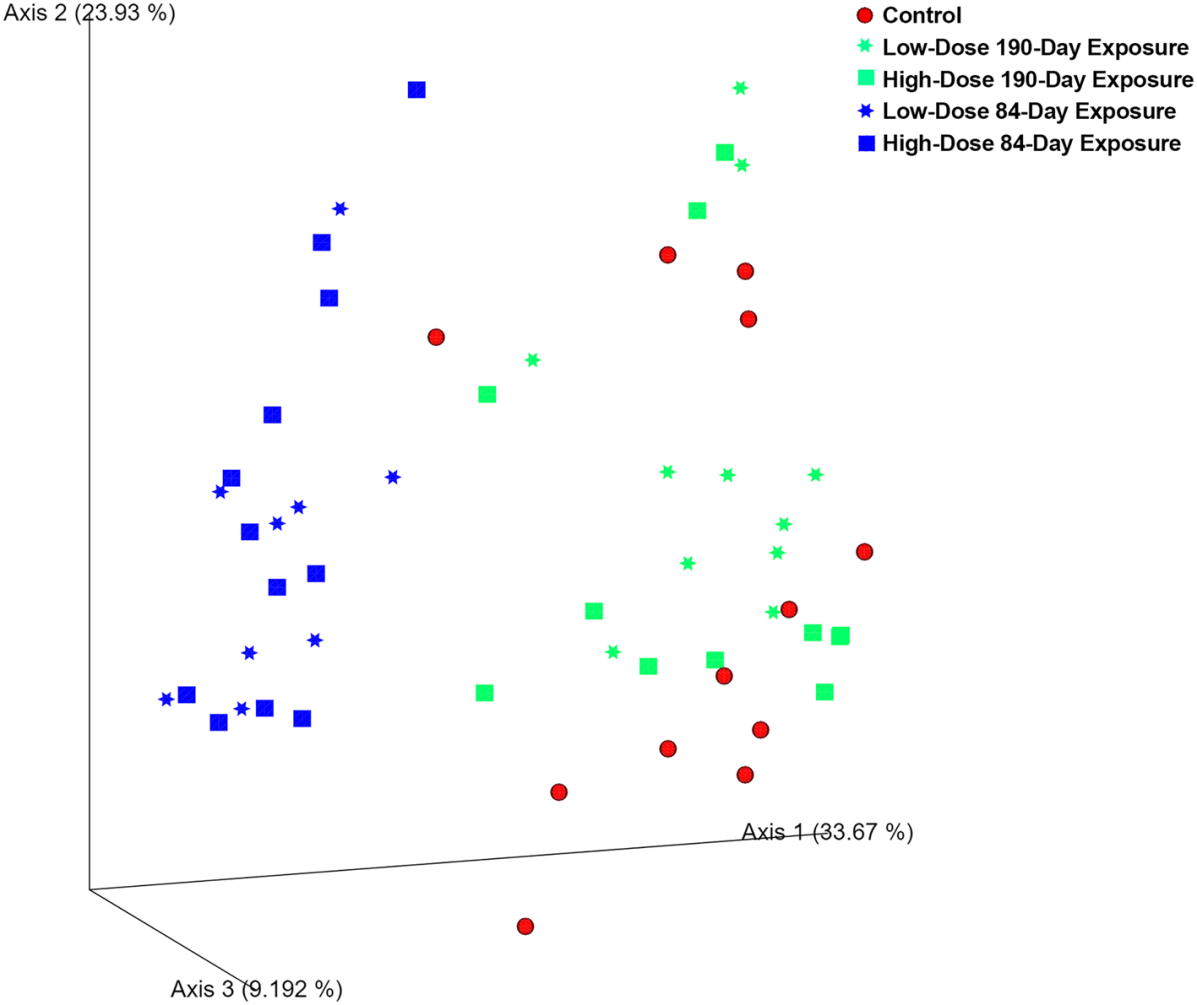
Ordination of weighted Unifrac distances for 16S ASVs among soybean root-associated bacterial community samples. Sequences diverged along axis 1 based on exposure duration. Samples exposed for 84 Days were significantly different from controls and samples exposed for 190 Days, based on pairwise PERMANOVA with q-values ≤ 0.05 (see Supplementary Table S3).

In contrast to the soil interaction time driver of alpha diversity changes, both CeO_2_ NP dose and exposure duration were the treatment variables associated with beta diversity shifts. The distances between low-dose and control ASVs were significantly greater than those within either group. Likewise, high-dose samples were significantly different from controls (q-values ≤ 0.05, Supplementary Table S3). Regarding exposure duration, there was prominent divergence of weighted Unifrac distances between ASVs from 84-day samples and others. Ordination of these distances shows that sequences primarily diverged along axis 1 based on exposure duration, which explained 33.7% of the variance among samples (Fig. 3). Weighted Unifrac measurements allow quantitative comparisons of microbial community composition dissimilarity among samples. That dissimilarity is based on the sum of branch lengths from ASV phylogenetic trees, which are weighted by relative abundance of variants. Each of the pairwise distances among 190-day, 84-day and control samples were significantly different than those within groups (Supplementary Table S3). Between the treatment variables, weighted Unifrac distances were more highly correlated with exposure duration than dose (Table 2).

**Table 2:** Ranked correlations of soil CeO_2_ NP dose and exposure duration with soybean root-associated bacterial 16S rRNA gene ASV weighted Unifrac distances.

Variables	Size	Correlation
CeO_2_ NP Exposure Duration	1	0.28
CeO_2_ NP Dose, Exposure Duration	2	0.14

Weighted Unifrac ordination of ASV divergence with respect to rhizosphere, rhizoplane, and root compartments can be seen along axis 2 in Supplementary Fig. S2, and it explained 23.9% of the variance among samples. Beta diversity group significance tests showed that root ASV distances were significantly different from those in both rhizoplane and rhizosphere (q-values ≤ 0.05, Supplemental Table S3). Additional weighted Unifrac group significance test results for compartment, dose, and exposure are shown in Supplemental Table S4 and Fig. S3. At this level of granularity, 69 of the 105 pair-wise comparisons had the lowest q-values of 0.055, and 60% of those came from comparisons with rhizoplane samples. Interestingly, there was a trend of greater distances between 84-day exposed communities and controls across all compartments, especially in the rhizoplane and rhizosphere.

**Figure 4:**
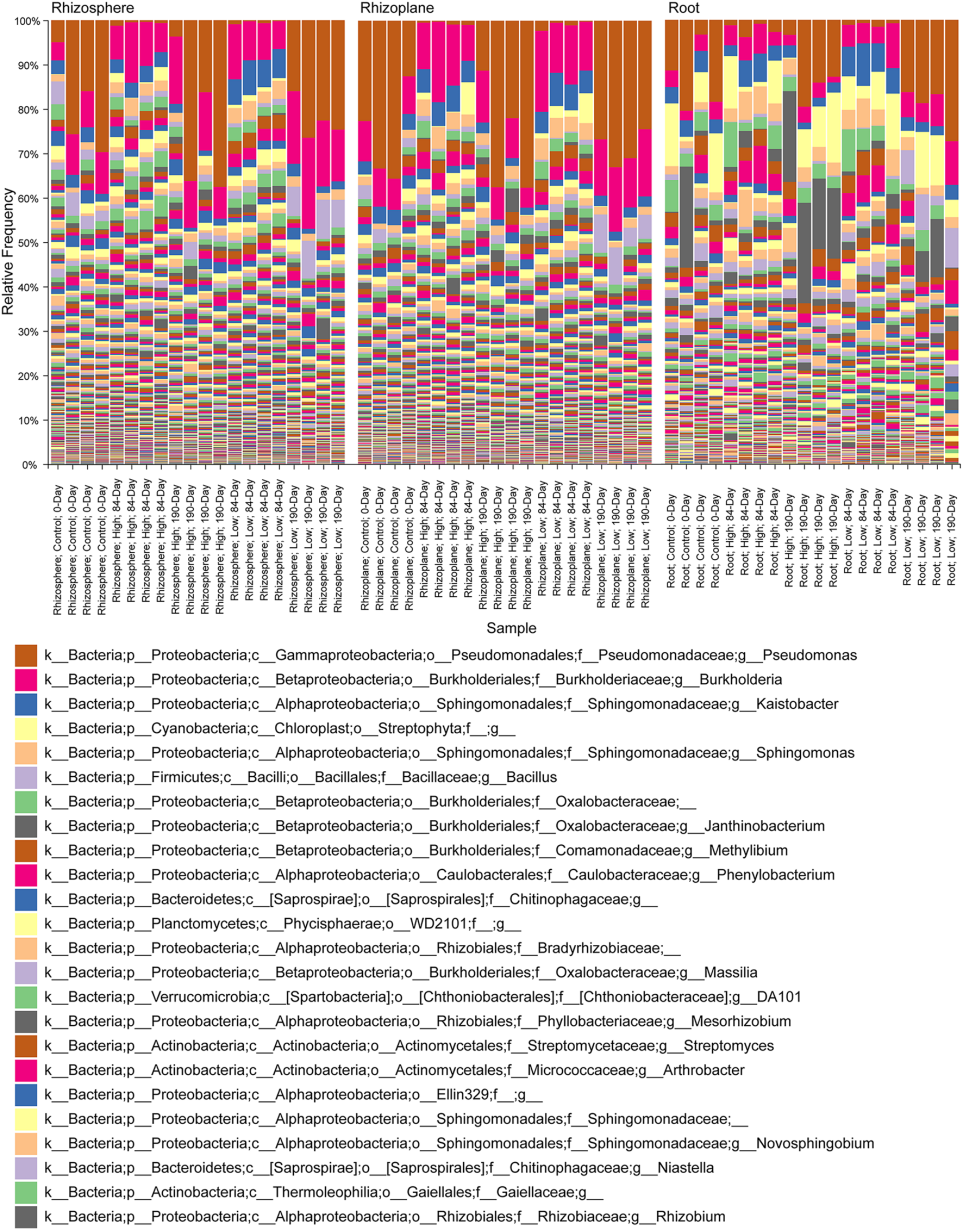
Relative frequency bar plot of bacterial taxa classified from 16S ASV data. The bars are seperated by rhizosphere, rhizoplane, and root compartments. Bars are shown for each of the 4 replicates/experimental group/compartment. Taxonomic identifications were made to the genus-level, where possible. The legend is restricted to the top 24 most frequent taxa.

**Figure 5:**
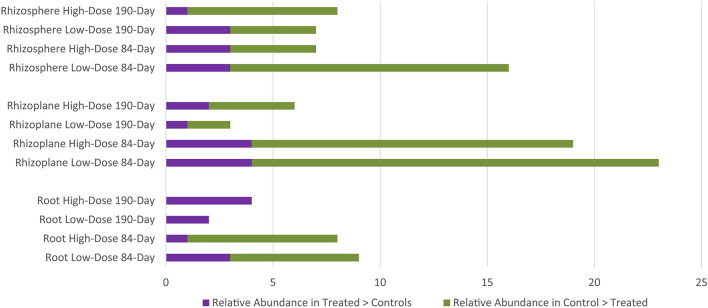
Counts of differentially abundant ASVs compared to controls were highest for rhizoplane low-dose soil samples exposed for 84 Days. Differential abundance was based on ANCOM Wilcoxon sum of the signed ranks for ASVs with corrected FDR ≤ 0.05.

A relative frequency bar plot of bacterial taxa identified from our 16S rRNA gene sequences is shown in Fig. 4, which suggests that primary effects on the bacterial communities were associated with 84-day exposure to CeO_2_ NPs. For example, the relative frequency of *Pseudomonas* sp. ASVs were < 5% for all 84-day samples, while in other groups they often exceeded 15%. We identified ASVs with significantly altered differential abundance in treated samples vs. controls, and our stringent filtering criteria restricted those to only the top 73 ASVs (Fig. 5, Supplementary Table S5 summary and taxonomic IDs for ASVs). The results of our analyses clarify the interactive effects of NP dose and exposure duration on the plant-mediated bacterial community responses and spatial distribution of impacts nested within the compartments. Specifically, the first and second highest counts of differentially abundant ASVs were from rhizoplane 84-day samples, with a maximum of 23 for the low-dose treatment. The rhizoplane is the compartment where root exudates would also be at their highest concentrations within the soil. In addition, 84-day low-dose samples had the highest counts of differentially abundant ASVs in all 3 compartments. The shorter NP-soil interaction time coinciding with presence of plants resulted in non-monotonic responses to dose in the rhizosphere, rhizoplane, and root, which mirror the patterns within the soybean stem and leaf mass data described above. In contrast, the counts of differentially abundant ASVs from 190-day samples were muted compared to those exposed for 84 days, and interestingly, they showed positive dose–response relationships.

Taken together, our findings suggest that plant-mediated effects to the microbial communities increase along a root exudate concentration/impact gradient within the soil, leading to peak effects within the rhizoplane, like previous results with canola^37^. Although we did not sample root exudates, the results are consistent with the putative sequence of events described above. It is also possible that non-monotonic developmental increases in the 84-day exposed soybean plants extend to quantitative and/or qualitative differences in expression of root exudates, which would explain the parallel effects within the compartments. However, mechanisms linking those changes are unknown.

Non-monotonic soybean responses to CeO_2_ NP exposure were previously modeled as the interaction between two, individually competing monotonic, dose–response processes^26^. The first for the plant and the second for the symbiotic bacteroids (modified cells formed by symbiotic bacteria in a root nodule of leguminous plants). In the model, symbionts are assumed to be either parasites that do not fix N_2_ or N_2_ fixing mutualists, depending on environmental conditions such as nitrogen availability from the soil. While the simulation predicts non-monotonic responses to CeO_2_ NP exposure when symbionts have parasitic interactions with plants^26^, some points of clarification are in order regarding how plant-symbiont interactions might change under host stress. Naturally occurring strains of rhizobia within species, such as those in the genus *Bradyrhizobium*, can have genetic differences that effect their nodulation (Nod) and nitrogen fixation (Fix) capacity. Combinations of these traits contribute to different endosymbiont-host strategies^70^. For example, mutualistic strains form and infect legume root nodules (Nod^+^), then provide their hosts with nitrogen (Fix^+^). Parasitic strains infect legumes, but fix little or no nitrogen (Nod^+^/Fix^−^). Whereas nonsymbiotic strains are unable to infect legumes at all (Nod^−^/Fix^−^)^71^ and references therein. Coinfections of hosts that include mutualistic and parasitic strains are common and can result in sectoring of strains within individual nodules. Host plant sanctions against parasitic strains can potentially operate on several levels ranging from whole nodules down to cell autonomous senescence within nodules^72,73^. It is possible that some rhizobia could have conditional strategies, such as modulating their N_2_ fixation rate^70^. However, to our knowledge no cases of facultative N_2_ fixation have been reported for *B. japonicum* strains in soybean nodules. On the other hand, studies on bacterial symbiotic transitions have demonstrated that gains and losses of genetic loci for symbiosis in bacterial lineages occur on evolutionary time scales^74,75^. From that perspective, it is likely that mixtures of parasitic and mutualistic strains are present in host plants when the uptake of CeO_2_ NPs or Ce^3+^ cations occurs in the roots. Afterwards, oxidative stress responses in the plants might accelerate highly localized host sanctions against lingering parasitic Fix^−^ strains in nodules and thereby shift the mix towards mutualists. Future modeling efforts of non-monotonic responses in this system that account for such scenarios are needed.

Testing for stress induced changes in proportions of Fix^+^ and Fix^−^ *B. japonicum* symbiont strains potentially behind non-monotonic soybean responses was beyond the scope of this paper. While we detected Bradyrhizobiaceae ASVs in all compartments (Fig. 4, Supplementary Fig. S4), differential abundance was only seen in rhizoplane high-dose 190-day samples compared to controls (Supplementary Table S5). Additional sequencing data from phylogenetically informative loci besides 16S will be needed to resolve strain level changes in abundance. Shifts in the relative abundance of parasitic and mutualistic *B. japonicum* bacteroids in nodules could have been expedited through feedback from plant stress responses to cerium uptake and host cell autonomous senescence sanctions^73^.

### Metabolic pathway analyses

**Figure 6:**
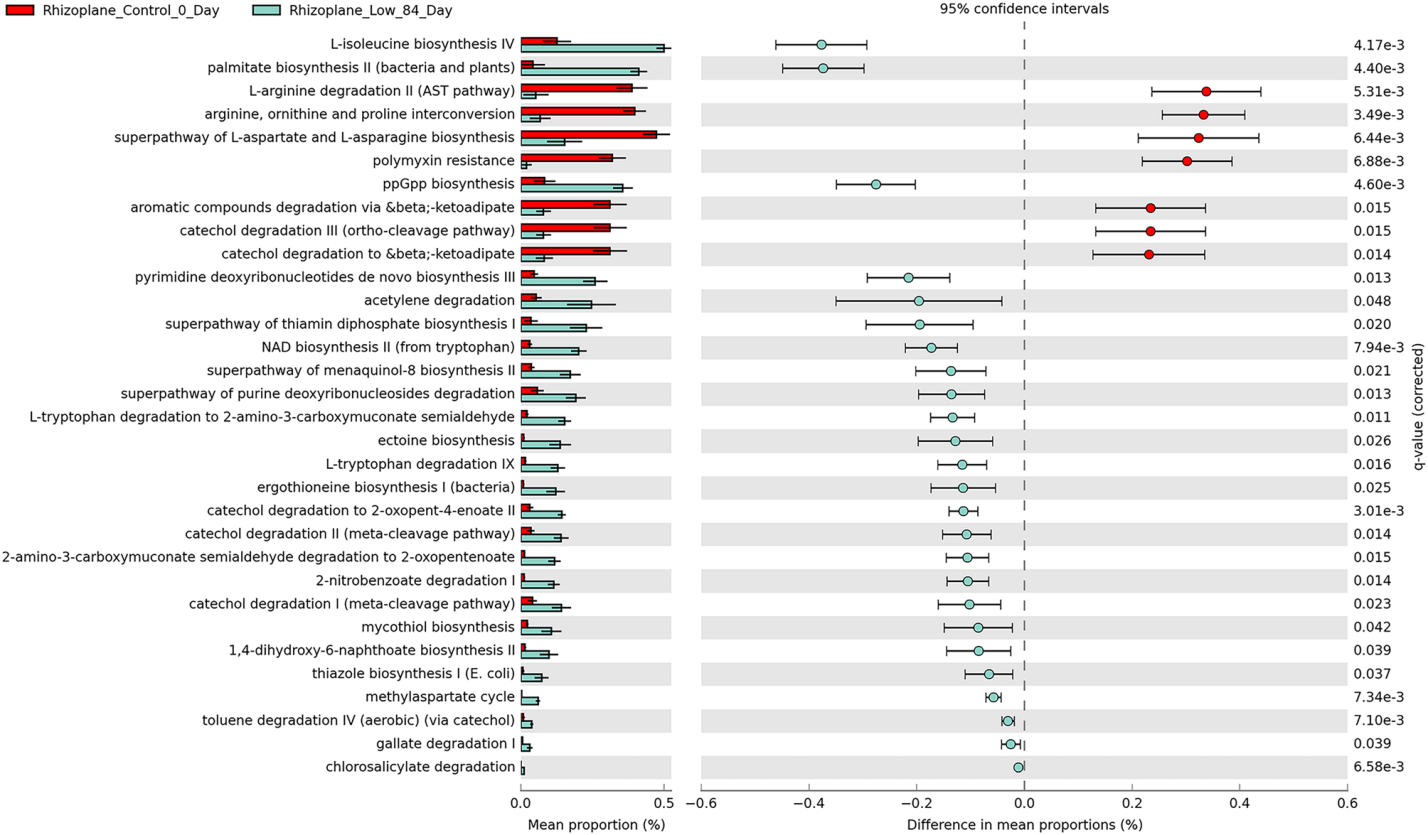
Inferred MetaCyc pathways for differentially abundant ASVs from rhizoplane low-dose, samples exposed for 84 Days, sorted by effect size. Pathways were identified with PICRUSt2 based on Welch’s two-way t-test, with Benjamini–Hochberg multiple test correction, Confidence Interval = 0.95. Pathways were filtered for q ≤ 0.05 and to remove those with effect sizes < 3.

We made bioinformatic inferences of MetaCyc metabolic pathway shifts from our differentially abundant 16S rRNA gene ASVs, comparing predicted functional changes in bacterial communities between the experimental groups. Significantly enriched and depleted pathways were only detected from our 84-day exposed samples (CI = 0.95, q ≤ 0.05, and effect size ≥ 3). For the low-dose 84-day cases, there were 14, 32, and 24 differentially abundant pathways in the rhizosphere, rhizoplane, and root compartments, respectively (Fig. 6, Supplementary Figs. S5 and S6). At most, 18 were shared between rhizoplane and roots and only 5 were common in all compartments (Supplementary Fig. S7).

**Figure 7:**
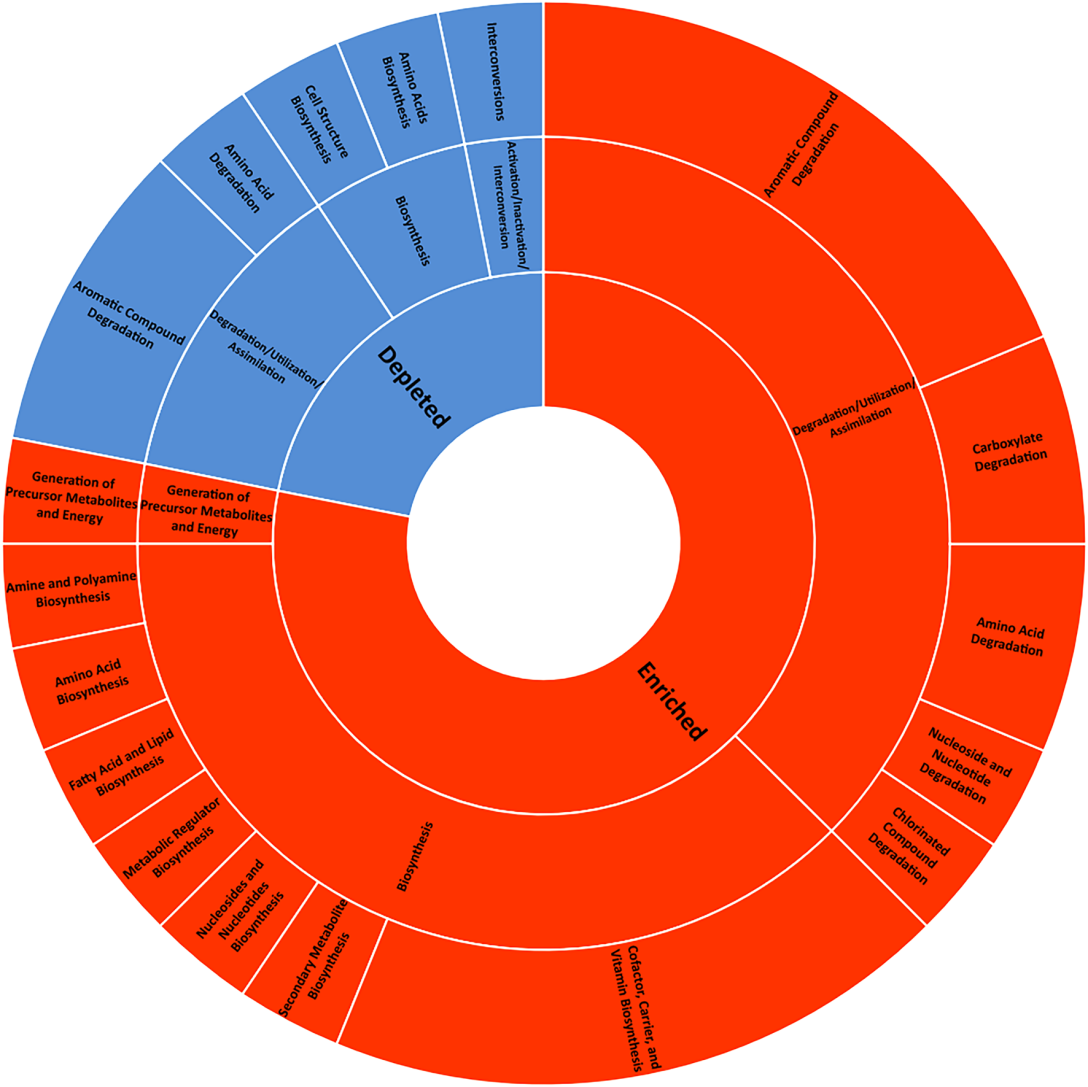
Overview of MetCyc pathway hierarchies for differentially abundant rhizoplane low-dose, samples exposed for 84 Days. Enrichment of inferred pathways was primarily related to biosynthesis and degradation/utilization/assimilation.

For rhizoplane and roots, 78% and 67% of pathways were enriched for treated samples, respectively. Whereas the pathways for rhizosphere treated samples were evenly split between those that were enriched or depleted. In all cases, metabolic pathways were primarily related to biosynthesis and degradation/utilization/assimilation, rather than responses to metals or oxidative stress (Fig. 7, Supplementary Figs. S8 and S9).

While subtle direct effects of the 84-day CeO_2_ NP exposure on the functions of bacterial communities cannot be excluded, the absence of detectable changes in pathways related to metal binding or stress responses to metals suggest that plant-mediated effects are dominant in this context. There were no significantly enriched or depleted pathways for 190-day exposed samples, so potential direct effects of the NPs set in place during the 3 months of interactions before planting will require further investigation.

## Conclusions

We found support for both CeO_2_ NP soil concentration and exposure duration effects on soybean development. The plants were impacted by the range of environmentally relevant soil NP doses under the short and long exposure times. Among the changes, there were non-monotonic dose-responses in plant development, which were exposure duration specific. The results suggest that the low-dose 84-day exposures initiated at planting were more stressful than other treatments to the above ground growth on soybeans. During the first 3 months of the 190-day exposure, CeO_2_ NPs were likely transformed by soil and microbial interactions and became less toxic to seedlings when they were planted.

Likewise, our results indicate that interactions between dose and exposure duration altered impacts to root-associated bacterial communities. The 190-day exposure was associated with lowered alpha diversity of bacterial ASVs compared to controls. The beta diversity distances between 84-day exposed samples were prominently divergent from all others. For all compartments, the largest counts of differentially abundant ASVs came from low-dose 84-day exposed samples.

We also report evidence for plant-mediated responses in the root-associated bacterial communities. While the 106-day NP-soil interaction time before planting may have been sufficient to mitigate effects on sensitive taxa, additional data are needed to test this hypothesis. Interestingly, we confirmed an impact gradient, with a maximum in rhizoplane microbial responses to 84-day exposure that point to effects from altered root exudates from plants. Those results showed non-monotonic responses that mirrored some changes in plants exposed to low-dose NPs. Furthermore, there were no indications of bacterial responses to metals or oxidative stress from our pathway analyses. Overall, our results suggest soybean-mediated responses of root-associated bacterial communities within the broader microbiome result from the integration of CeO_2_ NP dose and exposure duration impacts. The results highlight the importance of choices regarding applications of soil amendments such as biosolids containing CeO_2_ NPs or nano-enable formulations used in the cultivation of legumes and other crops.

### Supplementary Information

Supplementary Legends.

Supplementary Tables.

Supplementary Figures.

Supplementary Table S5.

## Data Availability

The 16S rRNA gene amplicon sequencing data pertaining to this manuscript are deposited in the National Center for Biotechnology Information under BioProject (PRJNA549519).

## Abbreviations

ANCOM: Analysis of compositions of microbiomes
ANOVA: Analysis of variance
ASV: Amplicon sequence variant
CeO_2_ NPs: Cerium oxide nanoparticles
FDR: False discovery rate
Fix: Nitrogen fixation
HSD: Honest significant difference
Nod: Nodulation
PERMANOVA: Permutational multivariate analysis of variance
PBS: Phosphate buffered saline
R6: Reproductive stage 6—full seed
R7: Reproductive stage 7—beginning maturity
SE: Standard error
