# Supplementary material for: CeO2 nanoparticle dose and exposure modulate soybean development and plant-mediated responses in root-associated bacterial communities
Source: Sci Rep. 2024 May 3;14:10231. doi: 10.1038/s41598-024-60344-8 (PMC11068890; doi:10.1038/s41598-024-60344-8)
Supplement: Supplementary file 1 — Supplementary Legends. [file 41598_2024_60344_MOESM1_ESM.pdf]

Supplementary Figure and Table Legend

Title

CeO_2 Nanoparticle Dose and Exposure Modulate Soybean Development and Plant-Mediated Responses in Root-Associated Bacterial Communities

Authors

Jay R. Reichman [a,b,c,*], Matthew R. Slattery [b], Mark G. Johnson [a], Christian P. Andersen [d], Stacey L. Harper [b,e]

[a] Pacific Ecological Systems Division, Office of Research and Development, US Environmental Protection Agency, Corvallis, Oregon 97333, United States

[b] Department of Environmental and Molecular Toxicology, Oregon State University, Corvallis, Oregon 97331, United States

[c] Department of Botany and Plant Pathology, Oregon State University, Corvallis, Oregon 97331, United States

[d] (Retired) Pacific Ecological Systems Division, Office of Research and Development, US Environmental Protection Agency, Corvallis, Oregon 97333, United States

[e] School of Chemical, Biological and Environmental Engineering, Oregon State University, Corvallis, Oregon 97331, United States

[*] Corresponding author.

E-mail address: reichman.jay@epa.gov

Supplementary Figure and Table Legend

Additional file 1:

Table S1. Physiochemical soil characterization. **Table S2.** Alpha diversity group significance for soybean root-associated bacterial 16S rRNA gene ASVs; Faith's phylogenetic diversity, Observed ASVs and Shannon Entropy. **Table S3**. Beta diversity group significance for soybean root-associated bacterial 16S rRNA gene ASV Bray-Curtis, Jaccard, unweighted Unifrac, and weighted Unifrac distances. **Table S4.** Weighted Unifrac compartment, dose, and exposure group significance for soybean root-associated bacterial 16S rRNA gene ASV distances.

Additional file 2:

Figure S1. Plant developmental parameter mean log plots. a) stem length, b) stem mass, c) leaves mass, d) shoots mass, e) pods count, f) pods mass, g) beans count, h) beans mass, i) nodules count, and j) nodules mass. Lower case letters indicate significant differences among means based on two-way ANOVA with HSD post hoc analysis (corrected $p \leq 0.05$). Error bars show ± 1 SE (N = 10). **Figure S2.** Ordination of 16S rRNA gene ASV weighted Unifrac distances colorized by microbial compartment. Root ASVs were significantly different from others based on pairwise PERMANOVA with q-values ≤ 0.05. **Figure S3.** Weighted Unifrac group significance distances to compartment controls for (a) rhizoplane, (b) rhizosphere, and (c) root**. Figure S4.** Mean Bradyrhizobiaceae ASV counts colorized by microbial compartment. 137 unique Bradyrhizobiaceae ASVs were detected across all compartments. **Figure S5.** Inferred MetaCyc pathways for differentially abundant ASVs from rhizosphere low-dose, 84-day exposed samples compared to controls, sorted by effect size. Pathways were identified with PICRUSt2 based on Welch's two-way t-test, with Benjamini-Hochberg multiple test correction, Confidence Interval = 0.95. Pathways were filtered for $q \leq 0.05$ and to remove those with effect sizes < 3. **Figure S6.** Inferred MetaCyc pathways for differentially abundant ASVs from root low-dose, 84-day exposed samples compared to controls, sorted by effect size. Pathways were identified with PICRUSt2 based on Welch's

two-way t-test, with Benjamini-Hochberg multiple test correction, Confidence Interval = 0.95. Pathways were filtered for $q \leq 0.05$ and to remove those with effect sizes < 3. **Figure S7.** Venn diagram for low-dose 84-day cases. There were 14, 32, and 24 differentially abundant pathways in the rhizosphere, rhizoplane, and root compartments, respectively. At most, 18 were shared between rhizoplane and roots and only 5 were in common for all compartments. **Figure S8.** Overview of MetCyc pathway hierarchies for differentially abundant rhizosphere low-dose, 84-day exposed samples. **Figure S9.** Overview of MetCyc pathway hierarchies for differentially abundant root low-dose, 84-day exposed samples.

Additional file 3:

Table S5. Summary of differentially abundant 16S rRNA gene ASVs. Differential abundance compared to controls was based on ANCOM Wilcoxon sum of the signed ranks for ASVs with corrected FDR ≤ 0.05. Tabs contain taxonomic identifications for differentially abundant ASVs for treatment combinations.
