# Supplementary material for: CeO2 nanoparticle dose and exposure modulate soybean development and plant-mediated responses in root-associated bacterial communities
Source: Sci Rep. 2024 May 3;14:10231. doi: 10.1038/s41598-024-60344-8 (PMC11068890; doi:10.1038/s41598-024-60344-8)
Supplement: Supplementary file 2 — Supplementary Tables. [file 41598_2024_60344_MOESM2_ESM.docx]

**Title**

CeO_2_ Nanoparticle Dose and Exposure Modulate Soybean Development and Plant-Mediated Responses in Root-Associated Bacterial Communities

**Authors**

Jay R. Reichman ^a,b,c,*^, Matthew R. Slattery ^b^, Mark G. Johnson ^a^, Christian P. Andersen ^d^, Stacey L. Harper ^b,e^

^a^ Pacific Ecological Systems Division, Office of Research and Development, US Environmental Protection Agency, Corvallis, Oregon 97333, United States

^b^ Department of Environmental and Molecular Toxicology, Oregon State University, Corvallis, Oregon 97331, United States

^c^ Department of Botany and Plant Pathology, Oregon State University, Corvallis, Oregon 97331, United States

^d^ (Retired) Pacific Ecological Systems Division, Office of Research and Development, US Environmental Protection Agency, Corvallis, Oregon 97333, United States

^e^ School of Chemical, Biological and Environmental Engineering, Oregon State University, Corvallis, Oregon 97331, United States

^*^ Corresponding author.

E-mail address: [reichman.jay@epa.gov](mailto:reichman.jay@epa.gov)

**Table S1. Physiochemical soil characterization**

| **Moisture** | | **Sand** | **Silt** | **Clay** | **pH** | **BpH** | **EC** |  | |
| --- | --- | --- | --- | --- | --- | --- | --- | --- | --- |
| 4.1% | | 63.8% | 20.1% | 16.1% | 6.59 | 7.03 | 0.393 |  | |
| **C** | | **N** | **C:N** | **Active C** | **NO_3_-N** | **NH_4_-N** | **P** | **S** | |
| 0.49% | | 0.035% | 13.9 | 135.1ppm | 54.6ppm | 0.9ppm | 10.8ppm | BQL | |
| **K** | | **Ca** | **Mg** | **Na** | **K** | **Ca** | **Mg** | **CEC** | |
| 49.0ppm | | 2032ppm | 589ppm | 178ppm | 0.13  meq/100g | 10.16  meq/100g | 4.91  meq/100g | 15.19  meq/100g | |
| **B** | | **Mn** | **Cu** | **Zn** | **Al** | **Co** | **Ni** | **Pb** | |
| 0.08ppm | | 22.0ppm | 1.84ppm | 1.26ppm | 1110ppm | 10.9ppm | 22.3ppm | 2.1ppm | |
| Moisture | | Gravimetric moisture as sample is received. All other data reported on a dry  matter basis | | | | | | | |
| Texture | | Determined with hydrometer method after cementing and flocculating agents  removed | | | | | | | |
| pH EC | | 1:1 water:soil ratio, measured on a Hanna benchtop meter | | | | | | | |
| BpH | | Sikora buffered pH for determining lime requirement | | | | | | | |
| Active  Carbon | | Readily oxidizable carbon measured by potassium permanganate reduction | | | | | | | |
| NO_3_, NH_4_ | | Extracted with 2M KCl, measured on Lachat autoanalyzer | | | | | | | |
| CEC | | Sum of bases estimation of Cation Exchange Capacity (CEC) | | | | | | | |
| K, Ca,  Mg, Mn, Zn, Cu, Fe | | Extracted with Mehlich 3 solution, measured on Agilent 5110 ICP-OES meq/100g units indicate millequivalents per 100 grams of soil | | | | | | | |
| B | | Extracted with 0.01M CaCl_2_ solution, measured on Agilent 5110 ICP-OES | | | | | | | |
| Na | | Extracted with 1M ammonium acetate solution, measured on Agilent 5110 ICP-  OES | | | | | | | |
| Al, Co,  Ni, Pb | | Microwave digestion for total nutrient content, measured on Agilent 5110 ICP-  OES | | | | | | | |
| BQL | | Below quantifiable limits | | | | | | | |

| **Table S2. Alpha diversity group significance for soybean root-associated bacterial 16S rRNA gene ASVs** | | | | | | | |  |  |
| --- | --- | --- | --- | --- | --- | --- | --- | --- | --- |
| **Krustal-Wallis (pairwise)** | |  |  |  |  |  |  |  |  |
|  | **Faith's Phylogenetic Diversity** |  |  | **Observed ASVs** |  |  | **Shannon Entropy** |  |  |
|  | **Group 1** | **Group 2** | **q-value** | **Group 1** | **Group 2** | **q-value** | **Group 1** | **Group 2** | **q-value** |
| **CeO_2_ NP Dose** | Control | High | 2.03E-01 | Control | High | 5.87E-02 | Control | High | 8.32E-01 |
|  |  | Low | 9.69E-02 |  | Low | 5.87E-02 |  | Low | 8.32E-01 |
|  | High | Low | 2.95E-01 | High | Low | 9.51E-01 | High | Low | 8.46E-01 |
|  |  |  |  |  |  |  |  |  |  |
| **CeO_2_ NP Exposure** | 190-Day | 84-Day | 4.23E-01 | 190-Day | 84-Day | 2.69E-01 | 190-Day | 84-Day | **1.90E-05** |
|  |  | Control | 1.20E-01 |  | Control | **2.99E-02** |  | Control | **2.36E-02** |
|  | 84-Day | Control | 1.87E-01 | 84-Day | Control | 1.49E-01 | 84-Day | Control | 2.62E-01 |
|  |  |  |  |  |  |  |  |  |  |
| **Microbial Compartment** | Rhizoplane | Rhizosphere | 8.71E-01 | Rhizoplane | Rhizosphere | 5.52E-01 | Rhizoplane | Rhizosphere | 4.49E-01 |
|  |  | Root | **5.60E-03** |  | Root | **7.25E-03** |  | Root | 1.16E-01 |
|  | Rhizosphere | Root | **5.60E-03** | Rhizosphere | Root | **8.07E-03** | Rhizosphere | Root | 1.16E-01 |

| **Table S3. Beta diversity group significance for soybean root-associated bacterial 16S rRNA gene ASV distances** | | | | | | | | |  |  |  |  |
| --- | --- | --- | --- | --- | --- | --- | --- | --- | --- | --- | --- | --- |
| **Pairwise PERMANOVA results (999 permutations)** | | | |  |  |  |  |  |  |  |  |  |
|  | **Bray-Curtis** |  |  | **Jaccard** |  |  | **Unweighted Unifrac** |  |  | **Weighted Unifrac** |  |  |
|  | **Group 1** | **Group 2** | **q-value** | **Group 1** | **Group 2** | **q-value** | **Group 1** | **Group 2** | **q-value** | **Group 1** | **Group 2** | **q-value** |
| **CeO_2_ NP Dose** | Control | High | **3.00E-03** | Control | High | **1.00E-03** | Control | High | **3.00E-03** | Control | High | **1.50E-02** |
|  | Control | Low | **1.65E-02** | Control | Low | **1.00E-03** | Control | Low | **3.00E-03** | Control | Low | **1.50E-02** |
|  | High | Low | **3.40E-02** | High | Low | **1.00E-03** | High | Low | **1.60E-02** | High | Low | 9.70E-02 |
|  |  |  |  |  |  |  |  |  |  |  |  |  |
| **CeO_2_ NP Exposure** | 84-Day | 190-Day | **5.00E-03** | 84-Day | 190-Day | **1.00E-03** | 84-Day | 190-Day | **1.00E-03** | 84-Day | 190-Day | **1.70E-02** |
|  | 84-Day | Control | **1.50E-03** | 84-Day | Control | **1.00E-03** | 84-Day | Control | **1.00E-03** | 84-Day | Control | **1.50E-03** |
|  | 190-Day | Control | **1.50E-03** | 190-Day | Control | **1.00E-03** | 190-Day | Control | **1.00E-03** | 190-Day | Control | **1.50E-03** |
|  |  |  |  |  |  |  |  |  |  |  |  |  |
| **Microbial Compartment** | Rhizoplane | Rhizosphere | 5.87E-01 | Rhizoplane | Rhizosphere | 3.27E-01 | Rhizoplane | Rhizosphere | 4.51E-01 | Rhizoplane | Rhizosphere | 1.14E-01 |
|  | Rhizoplane | Root | **1.50E-03** | Rhizoplane | Root | **1.50E-03** | Rhizoplane | Root | **1.50E-03** | Rhizoplane | Root | **1.50E-03** |
|  | Rhizosphere | Root | **1.50E-03** | Rhizosphere | Root | **1.50E-03** | Rhizosphere | Root | **1.50E-03** | Rhizosphere | Root | **1.50E-03** |

| **Table S4. Weighted Unifrac compartment, dose, and exposure group significance** | | |
| --- | --- | --- |
| **for soybean root-associated bacterial 16S rRNA gene ASV distances.** | |  |
| **Pairwise PERMANOVA results (999 permutations)** | |  |
|  |  |  |
| **Weighted Unifrac** |  |  |
| **Group 1** | **Group 2** | **q-value** |
| Rhizoplane Control 0-Day | Rhizoplane High 190-Day | 3.37E-01 |
| Rhizoplane Control 0-Day | Rhizoplane High 84-Day | 5.47E-02 |
| Rhizoplane Control 0-Day | Rhizoplane Low 190-Day | 5.47E-02 |
| Rhizoplane Control 0-Day | Rhizoplane Low 84-Day | 5.47E-02 |
| Rhizoplane Control 0-Day | Rhizosphere Control 0-Day | 3.04E-01 |
| Rhizoplane Control 0-Day | Rhizosphere High 190-Day | 2.78E-01 |
| Rhizoplane Control 0-Day | Rhizosphere High 84-Day | 5.47E-02 |
| Rhizoplane Control 0-Day | Rhizosphere Low 190-Day | 5.47E-02 |
| Rhizoplane Control 0-Day | Rhizosphere Low 84-Day | 5.90E-02 |
| Rhizoplane Control 0-Day | Root Control 0-Day | 5.47E-02 |
| Rhizoplane Control 0-Day | Root High 190-Day | 5.47E-02 |
| Rhizoplane Control 0-Day | Root High 84-Day | 5.47E-02 |
| Rhizoplane Control 0-Day | Root Low 190-Day | 5.47E-02 |
| Rhizoplane Control 0-Day | Root Low 84-Day | 2.33E-01 |
| Rhizoplane High 190-Day | Rhizoplane Low 190-Day | 5.47E-02 |
| Rhizoplane High 190-Day | Rhizoplane Low 84-Day | 5.47E-02 |
| Rhizoplane High 190-Day | Rhizosphere Control 0-Day | 1.14E-01 |
| Rhizoplane High 190-Day | Rhizosphere High 190-Day | 6.68E-01 |
| Rhizoplane High 190-Day | Rhizosphere High 84-Day | 5.47E-02 |
| Rhizoplane High 190-Day | Rhizosphere Low 190-Day | 5.47E-02 |
| Rhizoplane High 190-Day | Rhizosphere Low 84-Day | 5.47E-02 |
| Rhizoplane High 190-Day | Root Control 0-Day | 5.47E-02 |
| Rhizoplane High 190-Day | Root High 190-Day | 5.47E-02 |
| Rhizoplane High 190-Day | Root High 84-Day | 5.47E-02 |
| Rhizoplane High 190-Day | Root Low 190-Day | 5.47E-02 |
| Rhizoplane High 190-Day | Root Low 84-Day | 2.31E-01 |
| Rhizoplane High 84-Day | Rhizoplane High 190-Day | 5.47E-02 |
| Rhizoplane High 84-Day | Rhizoplane Low 190-Day | 5.47E-02 |
| Rhizoplane High 84-Day | Rhizoplane Low 84-Day | 5.08E-01 |
| Rhizoplane High 84-Day | Rhizosphere Control 0-Day | 5.47E-02 |
| Rhizoplane High 84-Day | Rhizosphere High 190-Day | 5.47E-02 |
| Rhizoplane High 84-Day | Rhizosphere High 84-Day | 5.47E-02 |
| Rhizoplane High 84-Day | Rhizosphere Low 190-Day | 5.96E-02 |
| Rhizoplane High 84-Day | Rhizosphere Low 84-Day | 1.29E-01 |
| Rhizoplane High 84-Day | Root Control 0-Day | 5.47E-02 |
| Rhizoplane High 84-Day | Root High 190-Day | 5.47E-02 |
| Rhizoplane High 84-Day | Root High 84-Day | 5.47E-02 |
| Rhizoplane High 84-Day | Root Low 190-Day | 5.47E-02 |
| Rhizoplane High 84-Day | Root Low 84-Day | 2.31E-01 |
| Rhizoplane Low 190-Day | Rhizosphere Control 0-Day | 5.47E-02 |
| Rhizoplane Low 190-Day | Rhizosphere High 190-Day | 5.47E-02 |
| Rhizoplane Low 190-Day | Rhizosphere High 84-Day | 5.47E-02 |
| Rhizoplane Low 190-Day | Rhizosphere Low 190-Day | 1.66E-01 |
| Rhizoplane Low 190-Day | Rhizosphere Low 84-Day | 5.47E-02 |
| Rhizoplane Low 190-Day | Root Control 0-Day | 5.47E-02 |
| Rhizoplane Low 190-Day | Root High 190-Day | 5.47E-02 |
| Rhizoplane Low 190-Day | Root High 84-Day | 5.47E-02 |
| Rhizoplane Low 190-Day | Root Low 190-Day | 5.47E-02 |
| Rhizoplane Low 190-Day | Root Low 84-Day | 2.30E-01 |
| Rhizoplane Low 84-Day | Rhizoplane Low 190-Day | 5.47E-02 |
| Rhizoplane Low 84-Day | Rhizosphere Control 0-Day | 5.47E-02 |
| Rhizoplane Low 84-Day | Rhizosphere High 190-Day | 5.47E-02 |
| Rhizoplane Low 84-Day | Rhizosphere High 84-Day | 5.47E-02 |
| Rhizoplane Low 84-Day | Rhizosphere Low 190-Day | 5.47E-02 |
| Rhizoplane Low 84-Day | Rhizosphere Low 84-Day | 7.84E-02 |
| Rhizoplane Low 84-Day | Root Control 0-Day | 5.47E-02 |
| Rhizoplane Low 84-Day | Root High 190-Day | 5.47E-02 |
| Rhizoplane Low 84-Day | Root High 84-Day | 5.47E-02 |
| Rhizoplane Low 84-Day | Root Low 190-Day | 5.47E-02 |
| Rhizoplane Low 84-Day | Root Low 84-Day | 2.31E-01 |
| Rhizosphere Control 0-Day | Rhizosphere High 190-Day | 1.12E-01 |
| Rhizosphere Control 0-Day | Rhizosphere High 84-Day | 5.47E-02 |
| Rhizosphere Control 0-Day | Rhizosphere Low 190-Day | 5.47E-02 |
| Rhizosphere Control 0-Day | Rhizosphere Low 84-Day | 5.47E-02 |
| Rhizosphere Control 0-Day | Root Control 0-Day | 5.47E-02 |
| Rhizosphere Control 0-Day | Root High 190-Day | 5.47E-02 |
| Rhizosphere Control 0-Day | Root High 84-Day | 5.47E-02 |
| Rhizosphere Control 0-Day | Root Low 190-Day | 5.47E-02 |
| Rhizosphere Control 0-Day | Root Low 84-Day | 2.33E-01 |
| Rhizosphere High 190-Day | Rhizosphere Low 190-Day | 5.47E-02 |
| Rhizosphere High 190-Day | Rhizosphere Low 84-Day | 5.47E-02 |
| Rhizosphere High 190-Day | Root Control 0-Day | 5.47E-02 |
| Rhizosphere High 190-Day | Root High 190-Day | 5.47E-02 |
| Rhizosphere High 190-Day | Root High 84-Day | 5.47E-02 |
| Rhizosphere High 190-Day | Root Low 190-Day | 5.47E-02 |
| Rhizosphere High 190-Day | Root Low 84-Day | 2.33E-01 |
| Rhizosphere High 84-Day | Rhizosphere High 190-Day | 5.47E-02 |
| Rhizosphere High 84-Day | Rhizosphere Low 190-Day | 5.47E-02 |
| Rhizosphere High 84-Day | Rhizosphere Low 84-Day | 6.77E-02 |
| Rhizosphere High 84-Day | Root Control 0-Day | 5.47E-02 |
| Rhizosphere High 84-Day | Root High 190-Day | 5.47E-02 |
| Rhizosphere High 84-Day | Root High 84-Day | 5.47E-02 |
| Rhizosphere High 84-Day | Root Low 190-Day | 5.47E-02 |
| Rhizosphere High 84-Day | Root Low 84-Day | 2.39E-01 |
| Rhizosphere Low 190-Day | Root Control 0-Day | 5.47E-02 |
| Rhizosphere Low 190-Day | Root High 190-Day | 5.47E-02 |
| Rhizosphere Low 190-Day | Root High 84-Day | 5.47E-02 |
| Rhizosphere Low 190-Day | Root Low 190-Day | 5.47E-02 |
| Rhizosphere Low 190-Day | Root Low 84-Day | 2.26E-01 |
| Rhizosphere Low 84-Day | Rhizosphere Low 190-Day | 5.69E-02 |
| Rhizosphere Low 84-Day | Root Control 0-Day | 5.47E-02 |
| Rhizosphere Low 84-Day | Root High 190-Day | 5.47E-02 |
| Rhizosphere Low 84-Day | Root High 84-Day | 5.47E-02 |
| Rhizosphere Low 84-Day | Root Low 190-Day | 5.47E-02 |
| Rhizosphere Low 84-Day | Root Low 84-Day | 2.33E-01 |
| Root Control 0-Day | Root High 190-Day | 7.14E-01 |
| Root Control 0-Day | Root High 84-Day | 7.67E-02 |
| Root Control 0-Day | Root Low 190-Day | 6.72E-02 |
| Root Control 0-Day | Root Low 84-Day | 4.22E-01 |
| Root High 190-Day | Root Low 190-Day | 2.33E-01 |
| Root High 190-Day | Root Low 84-Day | 2.78E-01 |
| Root High 84-Day | Root High 190-Day | 1.42E-01 |
| Root High 84-Day | Root Low 190-Day | 7.50E-02 |
| Root High 84-Day | Root Low 84-Day | 1.00E+00 |
| Root Low 84-Day | Root Low 190-Day | 2.78E-01 |
